# Emerging roles of m6A RNA modification in cancer therapeutic resistance

**DOI:** 10.1186/s40164-023-00386-2

**Published:** 2023-02-21

**Authors:** Wei-Wei Liu, Zhong-Yuan Zhang, Fei Wang, Hao Wang

**Affiliations:** 1 Department of Laboratory Medicine, The First Affiliated Hospital of USTC, Division of Life Sciences and Medicine, University of Science and Technology of China, Hefei, China; 2 School of Basic Medical Sciences, Shandong University, Jinan, China; 3 Department of Radiology, The First Affiliated Hospital of USTC, Division of Life Sciences and Medicine, University of Science and Technology of China, Hefei, China; 4 Neurosurgical Department, The First Affiliated Hospital of USTC, Division of Life Sciences and Medicine, University of Science and Technology of China, Hefei, China; 5 Core Unit of National Clinical Research Center for Laboratory Medicine, Hefei, China

**Keywords:** Cancer therapy, m6A, Chemoresistance, Immunotherapy

## Abstract

Marvelous advancements have been made in cancer therapies to improve clinical outcomes over the years. However, therapeutic resistance has always been a major difficulty in cancer therapy, with extremely complicated mechanisms remain elusive. N6-methyladenosine (m6A) RNA modification, a hotspot in epigenetics, has gained growing attention as a potential determinant of therapeutic resistance. As the most prevalent RNA modification, m6A is involved in every links of RNA metabolism, including RNA splicing, nuclear export, translation and stability. Three kinds of regulators, “writer” (methyltransferase), “eraser” (demethylase) and “reader” (m6A binding proteins), together orchestrate the dynamic and reversible process of m6A modification. Herein, we primarily reviewed the regulatory mechanisms of m6A in therapeutic resistance, including chemotherapy, targeted therapy, radiotherapy and immunotherapy. Then we discussed the clinical potential of m6A modification to overcome resistance and optimize cancer therapy. Additionally, we proposed existing problems in current research and prospects for future research.

## Introduction

Among more than 100 types of RNA modifications discovered in mammalian messenger RNA (mRNA) and noncoding RNA (ncRNA), m6A, which refers to methylation at the N6 position of adenosine, is reported as the most abundant type [1, 2]. Advanced techniques like m6A-seq and miCLIP-seq uncovered that m6A methylation targeted at consensus sequences like DRACH (D = G, A, or U; R = G or A; H = A, C, or U), mainly enriched in coding sequence (CDS) and 3’UTR region of mRNA, as well as other kinds of RNAs [1, 3]. m6A methylation has been verified to play a crucial role in determining RNA fates, including splicing, nuclear export, translation, stabilization and degradation, further regulating gene expression and cellular phenotypes [4]. m6A mediated-RNA epigenetic modification orchestrates many physiological activities, such as DNA repair, meiosis, tissue remodeling, and circadian rhythm, etc. [5, 6], while dysregulated m6A methylation participated in numerous pathologic processes, especially tumorigenesis, progression, metastasis and therapeutic resistance [7, 8].

In a global context, cancer remains the top threat to public health and imposes a severe economic burden on society [9]. Owing to the significant heterogeneity, cancer cells frequently display therapeutic resistance, which is the main obstacle for cancer treatment [10]. The rationales of chemoresistance can be basically categorized as reduced cellular accumulation, increased detoxification, increased DNA repair, decreased apoptosis, and autophagy [11]. And various studies have explicitly indicated that m6A modification could regulate drug resistance in these ways [12–14]. Cancer cells gradually develop radiotherapy resistance via enhancing DNA damage repair ability, altering expression of oncogene/tumor suppressors and cancer metabolism of cancer cell [15]. Correspondingly, there are evidences showing the regulatory roles of m6A methylation in these procedures. Moreover, therapeutic resistance appears as a thorny problem in immunotherapy, undoubtedly impeding its further development. Inspiringly, m6A modification not only exhibits the possibility to overcome the immunotherapy resistance, but also potentiate novel immunotherapy.

Overall, illuminating the influence of m6A methylation in therapeutic resistance has profound significance for exploring potential strategies to overcome resistance and develop more efficient therapeutic targets, ultimately optimizing the cancer therapy [4, 16]. In this review, we systematically summarize the latest studies, providing mechanistic insights into the formation of resistance under the regulation of m6A methylation, aiming at facilitating the development of potential therapeutic strategies. At same time, we point out the deficiency of current study and put forward some feasible solutions and promising research directions.

## m6A methylation and demethylation

The m6A methylation is mainly catalyzed by methyltransferase complex (MTC), a heterodimer constituted by Methyltransferase-like 3 (METTL3) and METTL14, in which METTL3 exerts catalytic function and METTL14 provides structural support and recognizes target RNA [17]. Wilms tumor 1-associated protein (WTAP), vir like m6A methyltransferase associated (VIRMA, also known as KIAA1429), RNA-binding motif protein 15 (RBM15), and zinc finger CCCH domain-containing protein 13 (ZC3H13) function as cofactors, localizing MTC to target sites and initiating methylation [5]. The demethylation is executed by two demethylases, Obesity-associated protein (FTO) and alkB homolog 5 (ALKBH5) [18, 19]. FTO is the first m6A “eraser” to be discovered and mainly effects mRNA stability, translation and splicing [20, 21]. As the homologue of FTO, ALKBH5 can modulate RNA splicing, export and degradation [22]. Particularly, they have different substrate affinities. Which one of m6A and m6Am is the preferential target of FTO remains controversial, while ALKBH5 exclusively demethylates m6A [23].

“Reader” can specifically recognize and bind to m6A sites to control RNA fate, realizing epigenetic regulation in gene expression and biological phenotypes. YT521-B homology (YTH) proteins, including YTHDC1–2 and YTHDF1–3, are extensively involved in RNA metabolism. YTHDC1 has been verified to facilitate RNA splicing, nuclear export and decay [24–26], and YTHDC2 contributes to enhancing translation efficiency and mRNA decay [27, 28]. YTHDF1 can facilitate translation initiation by interacting with eukaryotic initiation factor 3 (eIF3), YTHDF3 not only exerts a synergistic effect with YTHDF1 in promoting translation, but also cooperates with YTHDF2 to induce mRNA degradation [29, 30]. The insulin-like growth factor 2 mRNA binding protein family, IGF2BP1-3 recognize m6A targets via K homology domains, promoting RNA stability and translation [31]. The heterogeneous nuclear ribonucleoproteins (HNRNPs) are confirmed to elicit alternative splicing and mediate co-translational regulation [32]. Moreover, fragile X mental retardation proteins (FMRPs) is indispensable for RNA export [33].

"Writers", "erasers" and “readers” work jointly to catalyze, remove and recognize m6A methylation, establishing a reversible and dynamic equilibrium. Except for mRNA, ncRNA including tRNA, rRNA, miRNA, lncRNA and circRNA, can all be m6A-modified, which further influences RNA splicing, translation, maturation, transport, localization, and RNA–protein interactions.

## m6A and chemotherapy resistance

**Fig. 1:**
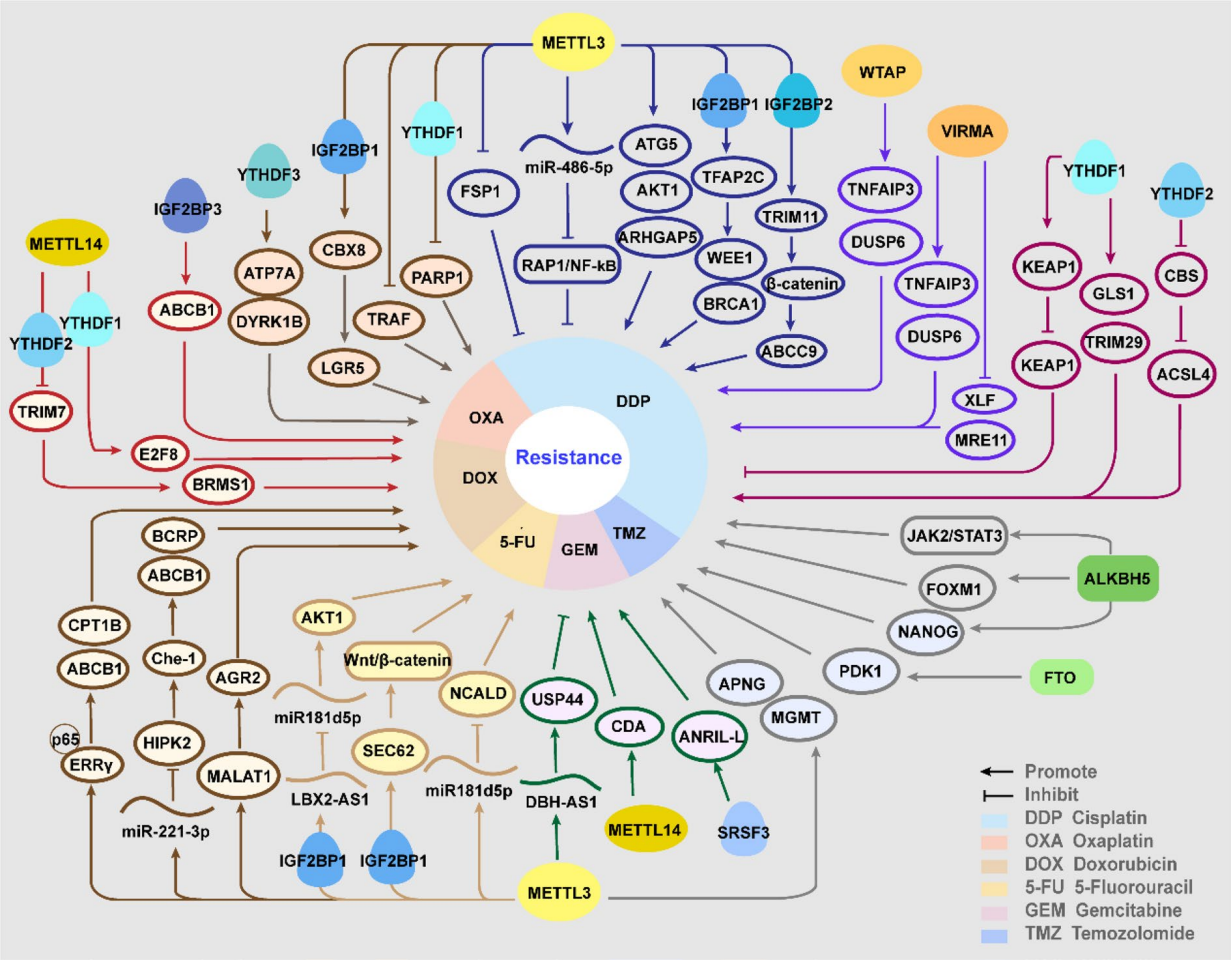
The role and molecular mechanisms of m6A regulators in chemotherapy resistance

Chemotherapy is one of the most potent clinical strategies for cancer treatment, especially in patients with advanced malignancy who cannot undergo surgery [34]. Resistance to chemotherapeutic agents greatly limits the overall treatment efficiency and develops into an increasingly austere clinical tissue [11]. Remarkably, growing evidences emphasized m6A modification as a potential determinant of tumor response to chemotherapy (Table 1; Fig. 1).

**Table 1:** Role of m6A regulator in chemotherapy

Drug	Regulator	Role	Level	Cancer		Target	Effect	References
Cisplatin	METTL3	Oncogene	High	NSCLC		AKT1	enhances expression of AKT1	[35]
Cisplatin	METTL3	Oncogene	NA	NSCLC		miR-486	promotes the maturation of pri-miR-486	[36]
Cisplatin	METTL3	Suppressor	Low	NSCLC		FSP1	enhances expression of FSP1	[37]
Cisplatin	YTHDF1	Suppressor	Low	NSCLC		KEAP1	Promotes transcription of KEAP1	[38]
Cisplatin	METTL3	Oncogene	NA	GC		ARHGAP5	Facilitates translation of ARHGAP5	[39]
Oxaliplatin	METTL3	Oncogene	High	GC		PARP1	stabilizes PARP1 mRNA via YTHDF1	[12]
Cisplatin	YTHDF2	Oncogene	NA	GC		CBS	Promotes degradation of CBS mRNA	[40]
Cisplatin	METTL14	Oncogene	NA	GC		c-Myc	stabilizes c-Myc mRNA via a potential reader, MSI2	[41, 42]
Oxaliplatin	METTL3	Oncogene	High	CRC		CBX8	stabilizes CBX8 mRNA via IGF2BP1	[43]
Oxaliplatin	METTL3	Oncogene	High	CRC		TRAF	Promotes TRAF mRNA degradation	[44]
Cisplatin	YTHDF1	Oncogene	High	CRC		GLS1	promoted translation of GLS1	[45]
Oxaliplatin	YTHDF3	Oncogene	High	CRC		ATP7A, DYRK1B	promotes translation of ATP7A and DYRK1B	[46]
Cisplatin	ALKBH5	Oncogene	High	EOC		JAK2	Promotes expression of JAK2	[47]
Cisplatin	YTHDF1	Oncogene	NA	OC		TRIM29	Promotes translation of TRIM29	[48]
Cisplatin	METTL3	Oncogene	High	Seminoma		TFAP2C	increased expression of TFAP2C via IGF2BP1	[49]
Cisplatin	METTL3	Oncogene	High	Seminoma		ATG5	Enhances expression of ATG5	[13]
Cisplatin	ALKBH5	Oncogene	High	OSCC		FOXM1, NANOG	Promotes expression of FOXM1 and NANOG	[50]
Cisplatin	FTO	Oncogene	High	OSCC		/	/	[51]
Cisplatin	METTL3	Oncogene	High	NPC		TRIM11	Stabilizes TRIM11 via IGF2BP2	[14]
Cisplatin	WTAP	Oncogene	High	NKTCL		TNFAIP3	Stabilizing TNFAIP3 mRNA	[52]
Cisplatin	WTAP	Oncogene	High	NKTCL		DUSP6	Increases stability of DUSP6 mRNA	[53]
Cisplatin	VIRMA	Oncogene	High	GCT		XLF, MRE11	Enhances expression of XLF and MRE11	[54]
Temozolomide	METTL3	Oncogene	High	GBM		MGMT and ANPG	Increases expression of MGMT and ANPG	[55]
Temozolomide	FTO	Oncogene	NA	GBM		PDK1	Reduces degradation of PDK1 mRNA	[56]
Temozolomide	ALKBH5	Oncogene	High	GBM		NANOG	Reduces degradation of NANOG mRNA	[57]
Gemcitabine	METTL3	Suppressor	Low	PC		lncRNA DBH-AS1	Promotes DBH-AS1 RNA stability	[58]
Gemcitabine	METTL14	Oncogene	High	PC		CDA	Stabilizes CDA mRNA	[59]
Gemcitabine	ALKBH5	Suppressor	Low	PDAC		WIF-1	Promotes transcription of WIF-1	[60]
Gemcitabine	SRSF3	Oncogene	High	PC		lncRNA ANRIL	Regulates alternative splicing ANRIL to increase the ANRIL-L isoform	[61]
5-Fluorouracil	METTL3	Oncogene	High	CRC	miR181b5p		Promotes the miR181b5p process by DGCR8	[62]
5-Fluorouracil	METTL3	Oncogene	High	CRC	LBX2-AS1		Stabilizes LBX2-AS1 via IGF2BP1	[63]
5-Fluorouracil	METTL3	Oncogene	High	CRC	SEC62		Promotes expression of SEC62	[64]
5-Fluorouracil	METTL3	Oncogene	High	CRC	P53		Promotes preferential splicing of p53 pre-mRNA, inducing p53 R273H mutant protein	[65]
Doxorubicin	METTL3	Oncogene	High	BC	MALAT1		METTL3 promotes MALAT1 expression level	[66]
Doxorubicin	METTL3	Oncogene	High	BC	miR-221-3p		Promotes process of miR-221-3p	[67]
Doxorubicin	METTL3	Oncogene	High	BC	ERRγ		Promotes expression of ERRγ	[68]
Doxorubicin	WTAP	Oncogene	High	BC	DLGAP1-AS1		Increases the stability of DLGAP1-AS1	[69]
Doxorubicin Cisplatin, Olaparib	YTHDF1	Oncogene	NA	BC	E2F8		YTHDF1 enhances the E2F8 mRNA stability	[70]
Doxorubicin	FTO	Oncogene	High	BC	STAT3		Promotes expression of STAT3	[71]
Doxorubicin	IGF2BP3	Oncogene	High	CRC	ABCB1		ABCB1 increases the stability of ABCB1 mRNA	[72]
Doxorubicin, Methotrexate	METTL3	Suppressor	Low	OS	TRIM7		Promotes degradation of TRIM7 via YTHDF2	[73]
Doxorubicin	METTL3	Oncogene	High	CML	PTEN		Promotes degradation of TRIM7 via PTEN	[74]
Bortezomib, Melphalan, Carfilzomib	METTL7A	Oncogene	NA	MM	LOC606724 and SNHG1		promotes enrichment of lncRNAs into exsomes	[75]
Arabinocytosine	METTL3	Oncogene	High	AML	MYC		Increases expression of MYC	[76]
Penicillin, Streptomycin	METTL3	Suppressor	Low	AML	AKT		Decreases expression of AKT	[77]
Dexamethasone	ALKBH5	Oncogene	NA	T-ALL	USP1		Increasing stability of USP1 mRNA	[78]

## Platinum drugs

The first generation of platinum drugs, cisplatin was approved by FDA for testicular cancer in 1965, then the second-generation carboplatin and third-generation oxaliplatin were utilized worldwide for multiple types of tumors [79]. However, resistance to platinum drugs is commonly seen in a short time, and m6A has been verified to regulate resistance to cisplatin (DDP) and oxaliplatin (OXA).

In non-small cell lung cancer (NSCLC), there is no unanimous conclusion about whether METTL3 facilitates or suppresses resistance to cisplatin. It was revealed that METTL3 contributed to insensitivity to cisplatin by enhancing AKT serine/threonine kinase 1 (AKT1) protein expression, and upregulated METTL3 in chemo-resistant NSCLC samples was positively related with poor survival [35]. However, Ling et al. found that propofol enhanced cisplatin sensitivity dose-dependently via promoting METTL3-mediated m6A methylation. Propofol augments m6A enrichment on pri-miR-486-5p to promote its maturation and further inactivates the Ras-associated protein1 (RAP1)-NF-kappaB (NF-κB) axis, and knockdown of METTL3 reversed the promoting effect [36]. Besides, METTL3 was negatively regulated by miR-4443, which is enriched in exosomes of cisplatin-resistant NSCLC. The decreased METTL3 further upregulated ferroptosis suppressor protein 1 (FSP1), reducing the ferroptosis induced by DDP and thus conferring chemoresistance [37]. Ablation of YTHDF1 mediates cisplatin resistance in NSLCC via decreasing translation of Kelch-like ECH-associated protein 1 (KEAP1) in an oxidative stress state induced by DDP, which in turn activates the antioxidant ROS clearance system aldo–keto reductases 1C1 (AKR1C1), thus inhibiting hypoxia-induced apoptosis. Moreover, the lower YTHDf1 level correlates with a worse clinical outcome for NSCLC patients [38].

In gastric cancer (GC), the previous studies confirmed in accordance that METTL3 augmented the resistance to platinum drugs. METTL3 was reported to methylate Rho GTPase activating protein 5 (ARHGAP5) mRNA to facilitate its translation, conferring resistance to chemotherapeutic drugs, including cisplatin, doxorubicin hydrochloride, and 5-fluorouracil (5-Fu) [39]. Additionally, in CD133 + GC stem cells, METTL3 play a decisive role in oxaliplatin resistance. In mechanism, the upregulated METTTL3 could enhance the stability of PARP1 mRNA by recruiting YTHDF1 to the 3'-UTR, and PARP1 contributes to repairing the drug-induced DNA damage [12]. YTHDF2 participates in the destabilization of Cystathionine β-synthase (CBS) mRNA induced by CBS mRNA-destabilizing lncRNA (CBSLR), downregulated CBS reduces the methylation of ACSL4, protecting GC cells from ferroptosis in vivo and in vitro, thus leading to a poorer response to chemotherapy [40]. Moreover, musashi RNA binding protein 2 (MSI2), was supposed to promote DDP resistance, acting as a potential m6A reader. In mechanism, LNC942 facilitates the expression of MSI2 by inhibiting its ubiquitination. MSI2 stabilizes c-Myc mRNA in an m6A-dependent manner [41]. Moreover, LNC942 was previously reported to stabilize downstream targets by recruiting METTL14, indicating the participation of m6A [42].

In colorectal cancer (CRC), METTL3 was proposed to be significantly upregulated and sustain oxaliplatin resistance. Mechanism studies elucidated that METTL3 could increase CBX8 mRNA stability dependent on IGF2BP1, and then CBX8 promotes the transcription of leucine rich repeat containing G protein-coupled receptor 5 (LGR5) to maintain cancer stemness and chemoresistance [43]. METTL3 triggered m6A modification on Tumor necrosis factor receptor-associated factors (TRAF) mRNA and promoted its degradation, thus suppressing necroptosis and reducing OXA sensitivity. Meanwhile, M2 tumor-associated macrophages (TAMs) conduced to OXA resistance formation by enhancing m6a methylation [44]. In DDP-resistant CRC cell, overexpressed YTHDF1 promoted protein synthesis of glutaminase 1 (GLS1) via binding to the 3' UTR of GLS1 mRNA. Suppression of YTHDf1-mediated glutamine metabolism sensitizes CRC cell to cisplatin in vitro and in vivo [45]. Moreover, YTHDF3 facilitates translation of several resistance biomarker genes, such as ATPase copper transporting alpha (ATP7A), dual-specificity Y-phosphorylation-regulated kinase 1B (DYRK1B), via recognizing the 5' UTR of these m6A-marked mRNAs and recruiting eIF3A.

In ovarian cancer (OC), ALKBH5 was upregulated and exerted an important role in the chemoresistance both in vivo and in vitro. ALKBH5 was increased via forming a loop with the upstream transcription factor homeobox A10 (HOXA10), then they jointly activated the Janus kinase 2 (JAK2)/signal transducer and activator of transcription 3 (STAT3) signaling pathway by m6A demethylation, thus promoting chemoresistance [47]. While Tripartite motif-containing protein (TRIM29) is supposed as an oncogene in OC related with the malignant characteristics, YTHDF1 binds to m6A sites on TRIM29 mRNA and facilitates its translation to enhance DDP resistance [48]. Additionally, comprehensive analysis of the transcriptome-wide m6A methylome of endometrioid OC showed that m6A enriched genes were significantly associated with resistance to platinum drug [80].

In seminoma, METTL3 was highly expressed in resistant seminoma cell and inhibition of METTL3 enhanced the response to cisplatin treatment. A recent study identified that METTL3-modified m6A methylation increased the expression of transcription factor-activating enhancer-binding protein 2C (TFAP2C), via IGF2BP1-mediated stabilization of its mRNA, further activated DNA repair genes WEE1 G2 checkpoint kinase (WEE1) and breast cancer type 1 (BRCA1) to promote resistance [49]. Additionally, overexpressed METTL3 confers resistance to DDP by enhancing autophagy. METTL3 could enhance the expression of ATG5, an autophagy elongation protein which increases autophagy to sustain cell viability and chemoresistance [13].

In oral squamous cell carcinoma (OSCC), the overexpression of ALKBH5 participates in the acquisition of resistance to DDP. In both resistant cell lines and clinical samples, highly expressed DDX3 directly elevates ALKBH5 level, compared with the sensitive counterparts [50]. Then ALKBH5 demethylated the transcripts of two transcription factors, forkhead box M1 (FOXM1) and Nanog homeobox (NANOG), to enhance their expression, both of which were previously reported as crucial for sustaining drug resistance [81, 82]. Also, it was verified that the upregulation of FTO was pivotal for arecoline‐induced OSCC stemness and chemoresistance to DDP [51].

In nasopharyngeal carcinoma (NPC), METTL3-mediated m6A modification was enriched in TRIM11 mRNA and stabilized it depending on IGF2BP2. TRIM11 facilitates the degradation of Daple by inducing ubiquitination, relieving the inhibition of Daple on Wnt/β-catenin signaling. Increased β-catenin directly bound to ABCC9 promoter to upregulate its expression, thus inducing chemoresistance via enhancing drug export [14]. Similarly, depletion of METTL3 in pancreas cancer results in higher sensitivity to several common anticancer drugs, such as gemcitabine, 5-FU and DDP in vitro, suggesting METTL3 as a potent target for improving therapeutic efficacy [83].

In bladder cancer, circ0008399 interacts with WTAP to facilitate the formation of MTC, stabilizing TNF alpha-induced protein 3 (TNFAIP3) mRNA by elevating its m6A modification level, which reduces the chemosensitivity to cisplatin [52]. Besides, in Nasal-type natural killer/T-cell lymphoma (NKTCL), WTAP was obviously upregulated and inactivate DDP by enhancing the mRNA stability of dual-specificity phosphatases 6 (DUSP6). Inhibition of WTAP decreased cell viability and reduced expression of drug resistance-associated protein MRP-1 and P-gp, indicating the therapeutic potential of WTAP in NKTCL [53]. VIRMA, another “writer”, was confirmed as an oncogene in Germ cell tumors (GCTs). VIRMA knockdown facilitated sensitivity to DDP, downregulated DNA damage repair players, XRCC4-like factor (XLF) and meiotic recombination 11 homolog 1 (MRE11) in a m6A-dependent way, thus enhancing DNA damage with increased expression of gamma histone variant H2AX (γH2AX) and growth arrest and DNA damage inducible alpha (GADD45A/B) [54].

## Temozolomide

Temozolomide (TMZ), another alkylating agent apart from platinum drugs, is the main first-line chemotherapeutic drug in the standard regimen of advanced gliomas, combined with radiotherapy [84]. However, the overall clinical efficacy of this regimen in Glioblastoma (GBM) remains disappointing, on account of inherent or induced resistance to TMZ treatment [55].

It was demonstrated that METTL3 level was elevated in GBM and promoted resistance to TMZ, both in vitro and in vivo. In mechanism, METTL3-mediated m6A modification enhances the expression of O6-methylguanine–DNA methyltransferase (MGMT) and alkylpurine–DNA–N-glycosylase (APNG) to repair DNA damage [55]. Moreover, Li et al. underlined the interplay of m6A modification and histone modification in forming resistance. TMZ treatment upregulated METTL3 through increasing chromatin accessibility at the METTL3 locus via SRY-box transcription factor 4 (SOX4). And METTL3 depletion could reduce m6A level on the enhancer of zeste 2 polycomb repressive complex 2 subunit (EZH2) mRNA, a histone modification-related gene, leading to mRNA degradation [85].

Intriguingly, the demethylase FTO was also reported to enhance the resistance to TMZ in GBM. FTO-mediated demethylation protected phosphoinositide dependent kinase-1 (PDK1) mRNA from degradation, while X-inactive specific transcript (JPX) significantly modulated the FTO/PDK1 interaction, leading to progression and chemoresistance of GBM [56]. Significantly, the FTO inhibitor R-2HG exhibits a synergistic effect with TMZ [86]. Besides, circ_0072083 could elevate ALKBH5 level by negatively regulating miR-1252-5p, and thus promote NANOG expression via reducing m6A modification-dependent degradation, while NANOG is an important biomarker for chemoresistance [57].

## Gemcitabine

Gemcitabine (GEM), a pyrimidine analogue, used widely in several tumors. Especially, its application has revolutionized the treatment of pancreatic cancer (PC), improving the survival rate up to 20% [87]. The chemoresistance to GEM, emerging as an urgent matter, has been revealed to be regulated by m6A modification.

METTL3 was supposed to play a positive role in regulating sensitivity to GEM. m6A methylation sustain RNA stability of DBH-AS1 in PC cells, which sensitizes PC cell to GEM by sequestering miR-3163 and thus upregulating USP44. Particularly, downregulation of DBH-AS1 was observed in GEM-resistant PC tissues and cells, and closely related with low expression of METTL3 [58]. Opposite to METTL3, the METTL14 was upregulated in GEM-resistant PC cells, induced by transcriptional factor p65 and downstream enhanced the expression of cytidine deaminase (CDA) to inactivate GEM. Suppression of METTL14 obviously enhance the sensitivity of GEM both in vivo and in vitro, indicating METTL14 as a promising target for improving chemotherapeutic effects in PC [59].

Interestingly, demethylase ALKBH5 also tend to promote sensitivity to GEM, and decreased ALKBH5 correlates with poor prognosis in pancreatic ductal adenocarcinoma (PDAC). The mechanism studies showed that overexpression of ALKBH5 could promote the transcription Wnt inhibitory factor 1 (WIF-1) via demethylation, thus inhibited Wnt pathway to sensitize cancer cells to chemotherapy [60]. Moreover, the increased m6A level on ANRIL recruited serine/arginine-rich splicing factor 3 (SRSF3) to regulates its splicing, facilitating the expression of ANRIL-L isoforms, which forms a complex with EZH2 and Ring1B to enhance DNA homologous recombination repair (HR), thus promote drug resistance [61].

## Impact on 5-FU resistance

5-FU is an analogue of uracil, incorporating into nucleic acids to interfere with nucleotide metabolism [88]. It has been widely involved in common chemotherapy regimens, including doublet PF (cisplatin and 5-FU), for many neoplasms treatment [89]. 5-FU is the first choice for CRC in the palliative and adjuvant settings, despite the unsatisfying response rate and frequent resistance [90].

Several studies have identified METTL3 as a driving force in the resistance to 5-FU. METTL3 was confirmed to inhibit the sensitivity through miR181b5p in cancer-associated fibroblasts (CAFs) secreted exosomes. The mechanistic study found that m6A modification enhanced the recognition of pri-miR181d by DiGeorge Syndrome Critical Region 8 (DGCR8) to promote the miR181b5p process. The increased miR181d5p interact with 3′-UTR of neurocalcin delta (NCALD) mRNA to suppress NCALD expression, inducing resistance to 5-FU in CRC cells [62]. It was revealed that METTL3 could enhance the stability of LBX2-AS1 dependent on IGF2BP1, upregulated LBX2-AS1 further increased AKT1 level by sponging miR-422a, correlated with poor response to 5-FU in CRC paitients [63]. Similarly, METTL3 promoted SEC62 expression via stabilizing its mRNA depending on IGF2BP1. SEC62 could protect β-catenin from degradation by directly binding to β-catenin and competitively inhibiting APC-β-catenin interaction, thus activate Wnt/β-catenin signaling to regulate stemness and chemoresistance to 5-Fu and oxaliplatin [64]. Moreover, methylation on p53 pre-mRNA catalyzed by METTL3 promotes a preferential splicing, inducing p53 R273H mutant protein, and giving rise to multidrug resistance in CRC cells, such as Oxa and 5-FU [65].

Nishizawa et al. proposed that inhibition of c-Myc-driven YTHDF1 transactivation suppresses CRC progression and triggers sensitization to anticancer drugs, such as OXA and 5-FU. And patients with high YTHDF1 levels were correlated with significantly poorer overall survival [91]. In accordance, another research showed that YTHDF1 was downregulated by miR-136-5p to suppress tumor progression and chemoresistance to 5-FU and OXA, while miR-136-5p was targeted by circPTK2 and declined in CRC cell lines and tissues [92].

## Adriamycin/Doxorubicin

Doxorubicin (DOX) is one of anthracycline antibiotics, exerting anti-tumor effects by intercalating into DNA and disrupting DNA repair subsequently [93], or through oxidative stress [94]. DOX remains one of the main treatments for early and advanced breast cancer (BC), and many other malignancies. Except for lethal cardiotoxicity, resistance is the major obstacle in chemotherapeutic approaches.

Several researches have elucidated different pathways for METTL3 to provoke resistance to DOX in BC. Li et al. have observed highly expressed METTL3 in DOX-resistant breast cancer cells. They supposed that METTL3 promoted expression of metastasis associated lung adenocarcinoma transcript 1 (MALAT1, which recruited E2F transcription factor 1 (E2F1) to activate transcription of anterior gradient 2 (AGR2), thus desensitize BC cells to DOX [66]. METTL3 could enhance pri-miR-221-3p maturation, miR-221-3p further suppresses HIPK2 and upregulates the downstream Che-1, which leads to increased multidrug resistance protein (MDR1) and breast cancer resistance protein (BCRP), indicating enhanced chemoresistance [67]. METTL3-mediated m6A modifications facilitate the expression of estrogen receptor related receptor γ (ERRγ) by triggering the splicing of ERRγ pre-mRNA, and ERRγ interacts with p65 to promote ABCB1 transcription, encoding P-gp to decrease the sensitivity to Taxol (Tax) and doxorubicin (Dox). ERRγ/p65 complex also binds to CPT1B promoter to increase its expression, accelerating fatty acid β-oxidation (FAO) and thus promoting resistance [68]. Moreover, de novo synthesis of fatty acids was supposed to be associated with chemoresistance in BC. Inhibition of FTO significantly attenuates fatty acid synthesis, which obviously sensitize BC cells to chemotherapy [95].

Also, attention has been given to other m6A modifiers about their influences on resistance to DOX in BC. WTAP enhanced the stability of lncRNA DLGAP1 antisense RNA 1 (DLGAP1-AS1) and overexpressed DLGAP1-AS1 promoted chemoresistance in vitro and correlated with unfavorable prognosis of BC patients. Furthermore, DLGAP1-AS1 motivated WTAP, by sponging miR-299-3p to relieve its suppression on WTAP, forming a feedback loop [69]. YTHDF1 was supposed as a novel target to deal with chemoresistance in BC and a putative tumor promotor [96]. The silico analysis showed that YTHDF1 was overexpressed in BC tissues, which facilitated DNA damage repair and resistance to Adriamycin, Cisplatin, and Olaparib. To achieve this, YTHDF1 enhances E2F8 mRNA stability dependent on METTL14, as E2F8 was identified to promote cell proliferation in BC by effecting G1/S phase transition [70]. Wang et al. reported that FTO and STAT3 were highly expressed in DOX-resistant BC cells and STAT3 directly binds to the promotor region of FTO to regulate its transcription. FTO knockdown could increase the chemosensitivity to doxorubicin and reverse the opposite effects of STAT3 overexpression [71].

Aside from breast cancer, m6A methylation was discovered to modulate DOX resistance in other cancers. Yang et al. figured out that upregulated IGF2BP3 in CRC could promote ABCB1 expression by increasing mRNA stability, thus induced resistance to DOX in vitro and in vivo [72]. In osteosarcoma (OS), ablation of METTL3 and METTL14 could upregulate the TRIM7 expression via inhibiting YTHDF2-medaited degradation. TRIM7, as a ubiquitin ligase, inhibits BRMS1 through ubiquitination, resulting in increased resistance to ADR and MTX in vitro and in vivo [73]. In DOX-resistant CML cells, LINC00470 promoted the degradation of phosphatase and tensin homolog (PTEN) mRNA via METTL3-modified methylation, which acts as a tumor suppressor by inhibiting Notch and PI3K‐AKT‐Mtor signaling. Knockdown of METTL3 rescued PTEN expression, thus inhibiting the leukemia cells viability and restoring the chemosensitivity [74].

## Other chemotherapeutic drugs

Wang et al. have demonstrated an unexplored role of adipocyte-derived exosomes to protect multiple myeloma (MM) cells against apoptosis induced by chemotherapy drugs such as bortezomib, melphalan, and carfilzomib. Herein, METTL7A promotes the enrichment of lncRNAs into exosomes to exert oncogenic roles, and the process is enhanced by MM cells through EZH2-mediated METTL7A methylation. This finding indicates a potential strategy to improve drug resistance by blocking this vicious exosome-mediated cycle [75]. In acute myeloid leukemia (AML), METTL3 was upregulated in resistant AML cells compared to the counterparts. METTL3 could promote chemoresistance to cytarabine (Ara-C) by facilitating expression of MYC [76]. However, bone marrow mesenchymal stem cells (MSCs) of AML have decreased global m6A levels and expressions of METTL3, and overexpressed METTL3 render MSCs sensitive to penicillin and streptomycin. Downregulated METTL3 increases AKT level and enhances adipogenesis, thereby contributing to chemoresistance [77]. In T-cell acute lymphoblastic leukemia (T-ALL), ALKBH5 could enhance the expression of ubiquitin-specific protease 1 (USP1) by stabilizing its transcripts. USP1 targeted at Aurora B and deubiquitinated it, facilitating resistance to dexamethasone in vitro and in vivo [78]. Using integrated bioinformatics analysis, the highly expressed RAB39B in DLBCL was revealed to be associated with 14 m6A regulators and resistance to several chemotherapy drugs including dexamethasone, doxorubicin, etoposide, vincristine, and cytarabine, as well as the poor survival in DLBCL patients [97]. A novel YTHDF3-based model was constructed to effectively predict the sensitivity of several therapeutic agents for breast cancer, including doxorubicin, paclitaxel, methotrexate, and vinorelbine [98]. Through silico analysis, researchers have supposed HNRNPC as a predictor of paclitaxel resistance in ovarian cancer [99]. Zhang et al. built an effective classifier to predict chemotherapy benefit of SCLC based on 7 m6A regulator-based. And in vitro experiments demonstrated that ZCCHC4, G3BP1, and RBMX may be potential therapeutic targets for overcoming chemoresistance in SCLCs [100].

## m6A and targeted therapy resistance

**Fig. 2:**
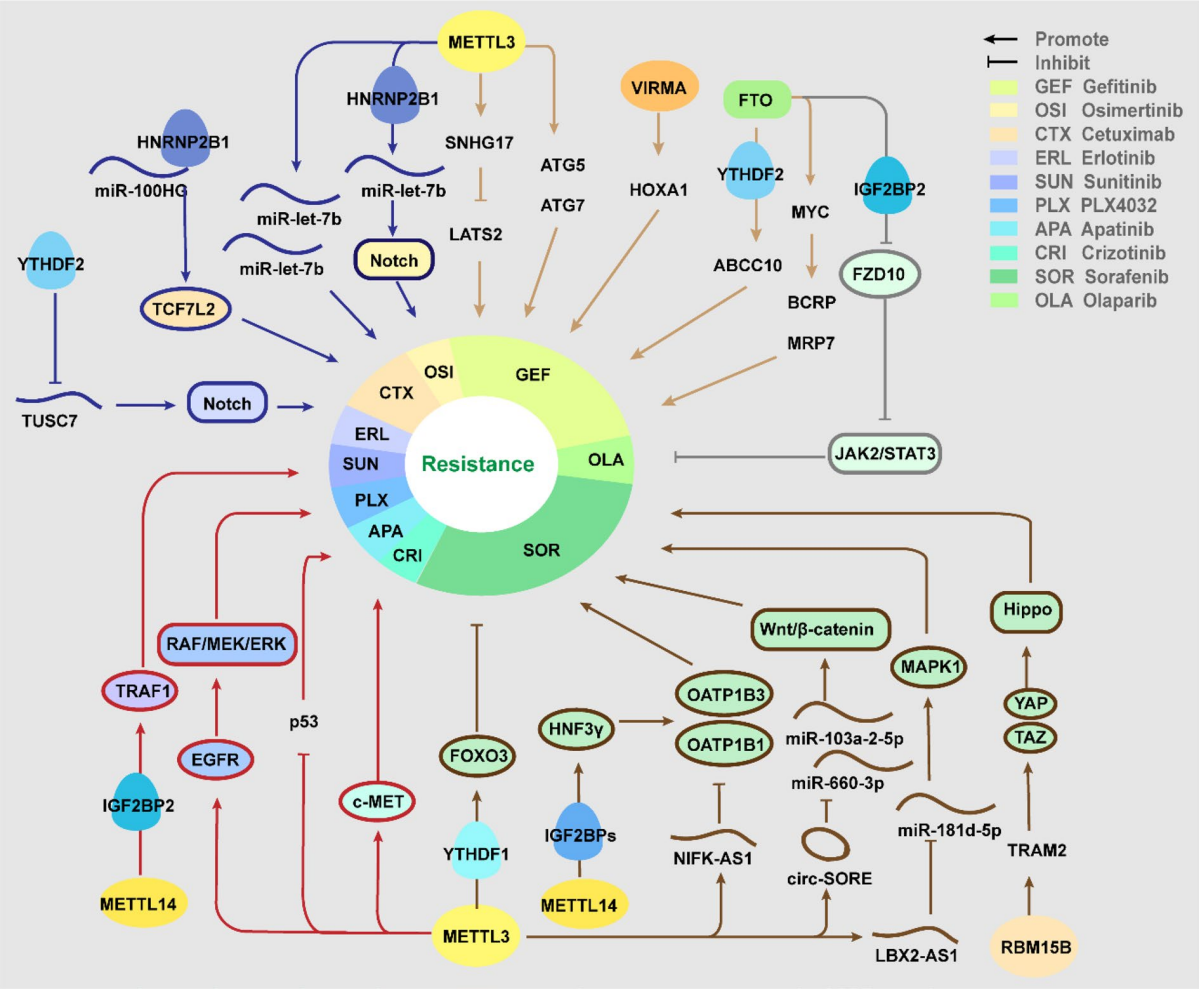
The role and molecular mechanisms of m6A regulators in Targeted therapy resistance

Targeted therapy is a kind of groundbreaking therapeutics by interfering with specific molecules to impede caner growth, progression and metastasis. A range of targeted medicines have been approved by FDA, displaying favorable clinical benefits in the treatment of various cancer types including liver, colorectal, lung, breast and ovarian cancers. Nevertheless, resistance to targeted therapy is frequently acquired, principally resulted from on-target mutations, bypass alterations, and cellular plasticity [101]. Meanwhile, m6A methylation has been confirmed to universally influence targeted molecules or pathways, regulating therapeutic resistance (Table 2, Fig. 2).

**Table 2:** Role of m6A regulator in targeted therapy

Drug	Regulator	Role	Level	Cancer	Target	Effect	Refs.
Sorafenib	METTL3	Oncogene	High	HCC	DUXAP8	Increases DUXAP8 RNA stability	[102]
Sorafenib	METTL3	Oncogene	High	HCC	NIFK-AS1	Upregulates NIFK-AS1 expression	[103]
Sorafenib	METTL3/14	Oncogene	High	HCC	SORE	Increases stability of SORE	[104]
Sorafenib	METTL3	Suppressor	Low	HCC	FOXO3	Stabilizes FOXO3 mRNA by YTHDF1	[105]
Sorafenib	METTL14	Suppressor	Low	HCC	HNF3γ	Increases HNF3γ mRNA stability via IGF2BPs	[106]
Sorafenib	RBM15B	Oncogene	High	HCC	TRAM2	Increases stability of TRAM2 mRNA	[107]
Gefitinib	METTL3	Oncogene	High	LUAD	lncRNA SNHG17	Increases SNHG17 RNA stability	[108]
Gefitinib	METTL3	Oncogene	High	LUAD	ATG5, ATG7	Upregulates expression of ATG5, ATG7	[109]
Gefitinib	KIAA1429	Oncogene	High	NSCLC	HOXA1	Increases HOXA1 mRNA stability	[110]
Gefitinib	FTO	Oncogene	High	NSCLC	ABCC10	Reduces the YTHDF2-mediated degradation of ABCB10	[111]
Gefitinib	FTO	Oncogene	High	NSCLC	MYC	promotes MYC expression	[112]
Erlotinib	YTHDF2	Oncogene	NA	LC	TUSC7	Promotes the degradation of TUSC7	[113]
Osimertinib	METTL3	Suppressor	Low	LUAD	Let-7b	Promotes the pri-Let-7b maturation via HNRNPA2B1	[114]
cetuximab	hnRNPA2B1	Oncogene	NA	CRC	TCF7L2	Increases stability of TCF7L2 mRNA	[115]
Cetuximab	METTL3	Oncogene	High	CRC	miR-100 and miR-125b	Reduces the miR-100 and miR-125b level and export	[116]
apatinib	METTL3	Oncogene	NA	GC	P53	Suppresses p53 activation	[117]
sunitinib	METTL14	Oncogene	NA	RCC	TRAF1	Increases TRAM1 mRNA stability via IGF2BP2	[118]
PLX4032	METTL3	Oncogene	NA	Melanoma	EGFR	Promotes translation of EGFR	[119]
crizotinib	METTL3, WTAP	Oncogene	NA	NSCLC	c-MET	Decrease expression of c-MET	[120]
Olaparib and Rucaparib	FTO, ALKBH5	Suppressor	Low		FZD10	Suppresses IGF2BP2-mediated stabilization of FZD10 mRNA	[121]

## Sorafenib

Sorafenib is a multiple-target tyrosine kinase inhibitor (TKI), which can inhibit Ras/Raf/MEK/ERK signaling pathways to restrain cancer cells proliferation, and target at receptors like vascular endothelial growth factor receptor (VEGFR) 2, platelet-derived growth factor receptor (PDGFR-β) to inhibit angiogenesis [122]. The application of sorafenib has significantly improved the outcomes of advanced HCC patients [123].

For both METTL3 and METTL14, there were contradictory findings about their regulatory roles in resistance to sorafenib. For the promotive role, the highly expressed METTL3 significantly enhanced the stability of lncRNA DUXAP8, which competitively binds to miR-584-5p via ceRNA mechanism, thus promotes MAPK1 expression and activates MAPK/ERK pathway, contributing to resistance acquisition in HCC [102]. METTL3 could upregulate NIFK-AS1, and NIFK-AS1 suppressed the expression of drug transporters Organic anion-transporting polypeptide 1B (OATP1B1) and OATP1B3 to inhibit sorafenib uptake, thus desensitize HCC cells. HCC patients with low level of NIFK-AS1 exhibited benefits to treatment [103]. In addition, m6A-circRNA interaction has been found in sustaining the resistance. METTL3/14 promoted circRNA SORE expression by increasing its stability, which acts as a sponge to sequester miR-103a-2-5p and miR-660-3p to activate the Wnt/beta-catenin pathway, contributing to resistance [104]. On the contrary, Lin et al. proposed that knockdown METLL3 can promote the resistance through FOXO3-mediated autophagy. METTL3 depletion attenuates the m6A level at 3′UTR of FOXO3 mRNA and depresses the YTHDF1-mediated stabilization, then downregulated FOXO3 induced the transcription of a series of autophagy-related genes to enhance autophagy in HCC cell lines [105]. Moreover, mechanism study showed that METTL14 could enhance HNF3γ mRNA stability via IGF2BPs, and thus induced transactivation of OATP1B1 and OATP1B3 expression, and reduced METTL14 in HCC cells contributes to developing resistance [106]. Except for the two principal writers, another methylase, RBM15B was also supposed to promote resistance to sorafenib in HCC. RBM15B interacts with the TRAM2 mRNA to enhance its stability, increased TRAM2 promotes the expression and activation of YAP and TAZ, activating the Hippo signaling pathway. Knockdown of RBM15B and TRAM2 significantly reversed the resistance, while overexpression exerted the opposite effects [107].

## Gefitinib

Overexpression of EGFR is ubiquitous in solid tumor and related with tumorigenesis, progression and invasion. EGFR-TKI treatment have provided tremendous therapeutic benefits for NSCLC patients with activating mutations of EGFR [124]. For LC patients with the in-frame deletion in EGFR-exon 19 or single-point mutation of exon 21, the standard first-line treatment is first-generation (gefitinib, erlotinib), or second-generation (afatinib) TKIs [125].

In NSCLC, existing evidences elucidated that METTL3 played a positive role in acquiring the resistance to gefitinib. METTL3 induced the upregulation of SNHG17 via stabilizing its mRNA, and SNHG17 recruited EZH2 to the promoter region of large tumor suppressor kinase 2 (LATS2) to suppress it expression, which was a well-known tumor suppressor involved in the Hippo cascade, then epigenetically repressed LATS2 promote resistance to gefitinib in LUAD [108]. Also, METTL3 can contribute to the resistance via increasing autophagy. The overexpressed METTL3 upregulated genes of autophagy pathway such as ATG5 and ATG7, while β-elemene inhibited the expression of METTL3 with highly selectivity, thus reversed the resistance via disrupting autophagy [109]. Besides, Tang et al. reported increased expression of “writer” KIAA1429 in resistant NSCLC cells, accompanied with high m6A enrichment. KIAA149 promote the resistance in vitro, via targeting the 3'-UTR of HOXA1 mRNA to enhance its stability in an m6A-depnendent way [110].

FTO has also been found to be upregulated in NSCLC and conducive to resistance to gefitinib. The gefitinib-resistant NSCLC patients exhibit increased FTO expression and decreased m6A level in the serum exosomes, and deletion of FTO significantly enhances the sensitivity. FTO facilitates ABCC10 expression via inhibiting the YTHDF2-mediated mRNA decay, promoting resistance in vitro and in vivo [111]. Significantly, the FTO inhibitor meclofenamic acid (MA) was identified to overcome drug resistance, via restraining FTO-mediated demethylation of m6A-modified MYC, and further downregulating the membrane drug efflux proteins, BCRP and MRP-7 [112].

## Other targeted therapies

Erlotinib is the first-generation EGFR inhibitor with similar molecular structures and pharmacologic mechanisms to gefitinib. In LUAD, YTHDF2-mediated inhibition of lncRNA TUSC7 provokes the resistance to erlotinib, as TUSC7 could sponge miR-146a and inhibit Notch signaling to decrease the cancer progression and improve sensitivity to erlotinib [113]. Osimertinib was the first 3rd-generation EGFR-TKIs to be approved for metastatic EGFR-mutant NSCLC. In osimertinib-resistant LUAD cells, metformin could upregulate METTL3 by promoting the bindings of DNA methyltransferase-3a/b (DNMT3a/b) to METTL3 promoter. Then METTL3-mediated m6A significantly facilitated pri-Let-7b maturation via NKAP and HNRNPA2B1, thus inactivating Notch signaling and re-captivating osimertinib treatment [114].

Cetuximab (CTX), a monoclonal antibody targeting EGFR, approved by FDA for treating metastatic CRC in 2004 [126]. The m6A reader HNRNPA2B1 was proposed to sustain resistance to CTX. Mechanistically, HNRNPA2B1 interacts with MIR100HG and recognizes the m6A sites of TCF7L2 mRNA to improve its stability, while TCF7L2 acts as an essential transcriptional coactivator of the Wnt/beta-catenin signaling [115]. Intriguingly, METTL3 was found to facilitate extracellular vesicle (EV)-mediated spread of the resistance by modulating miRNA metabolism. Knockdown of METTL3 decreased the cellular and extracellular levels of m6A-modified miRNAs, such as miR-100 and miR-125b, which were previously proved to induce resistance to CTX, and thus the EVs showed less capability of inducing the transfer of the resistance [116].

Apatinib, a selective TKI targeting at VEGFR-2 to anti-angiogenesis, applied in GC, HCC, and NSCLC and so on [127]. In HCC, suppression of METTL3 induced by S-adenosyl homocysteine or siRNA could activate p53, further sensitizing HCC cells to apatinib through apoptosis [117]. Sunitinib is a multi-target, anti-angiogenic TKI, which greatly improves the overall survival of metastatic RCC patients. The resistance to sunitinib in RCC was supposed to be linked with high expression level of TRAF1, as METTL14-mediated m6A modification enhances TRAF1 mRNA stability in a IGF2BP2-dependent manner [118].

PLX4032, a selective and potent inhibitor of BRAF V600E mutation, inhibits tumor growth in melanoma. Bhattarai et al. indicated that METTL3 could induce resistance to PLX4032 in melanoma via EGFR-mediated rebound activation. METTL3-mediated m6A modifications promoted the translation efficiency of EGFR mRNA, reactivating the RAF/MEK/ERK pathway to trigger resistance [119]. Crizotinib is a kinase inhibitor targeting c-MET/ALK/ROS1 used as the first-line therapy for NSCLC with ALK mutations. In ALK mutation-free and c-MET highly-expressed NSCLC cell lines, chidamide could enhance the sensitivity to crizotinib through decreasing expression of METTL3 and WTAP. Reduced m6A level on c-MET mRNA downregulated its expression, subsequently restored the response to crizotinib. And the c-MET ligand hepatocyte growth factor (HGF) could synergize with chidamide [120]. Poly(ADP-ribose) polymerase inhibitors (PARPi) such as Olaparib have demonstrated substantial therapeutic benefits for treating EOC patients with BRCA1/2 mutation [128]. In BRCA-mutated EOC cells, downregulated FTO and ALKBH5 elevated m6A level of FZD10 transcripts and stabilized it via IGF2BP2, further stimulated Wnt/β-catenin pathway, causing resistance to Olaparib and Rucaparib [121].

## m6A and radiotherapy resistance

Radiotherapy remains a mainstay of cancer treatment regimens, which kills cancer cells through damaging the DNA. Developing reliable predictive biomarkers for radio-resistance is significant for personalized radiation therapy [129]. Notably, METTL3 was previously verified to influence ultraviolet-induced DNA damage responses [130]. Depletion of METTL3 in pancreatic cancer cells lead to higher sensitivity to a series of anticancer therapy including irradiation (IR) [83]. In GBM, overexpressed METTL3 binds to SOX2 transcripts and sustains their stability, contributing to increased DNA repair, thus promoting the resistance of glioma stem-like cells to γ-irradiation [131]. Knockdown of METTL3 influenced the localization of DNA polymerase kappa to DNA damage sites in U2OS and HeLa cells, displaying insufficient repair and higher sensitivity to ultraviolet [130].

**Fig. 3:**
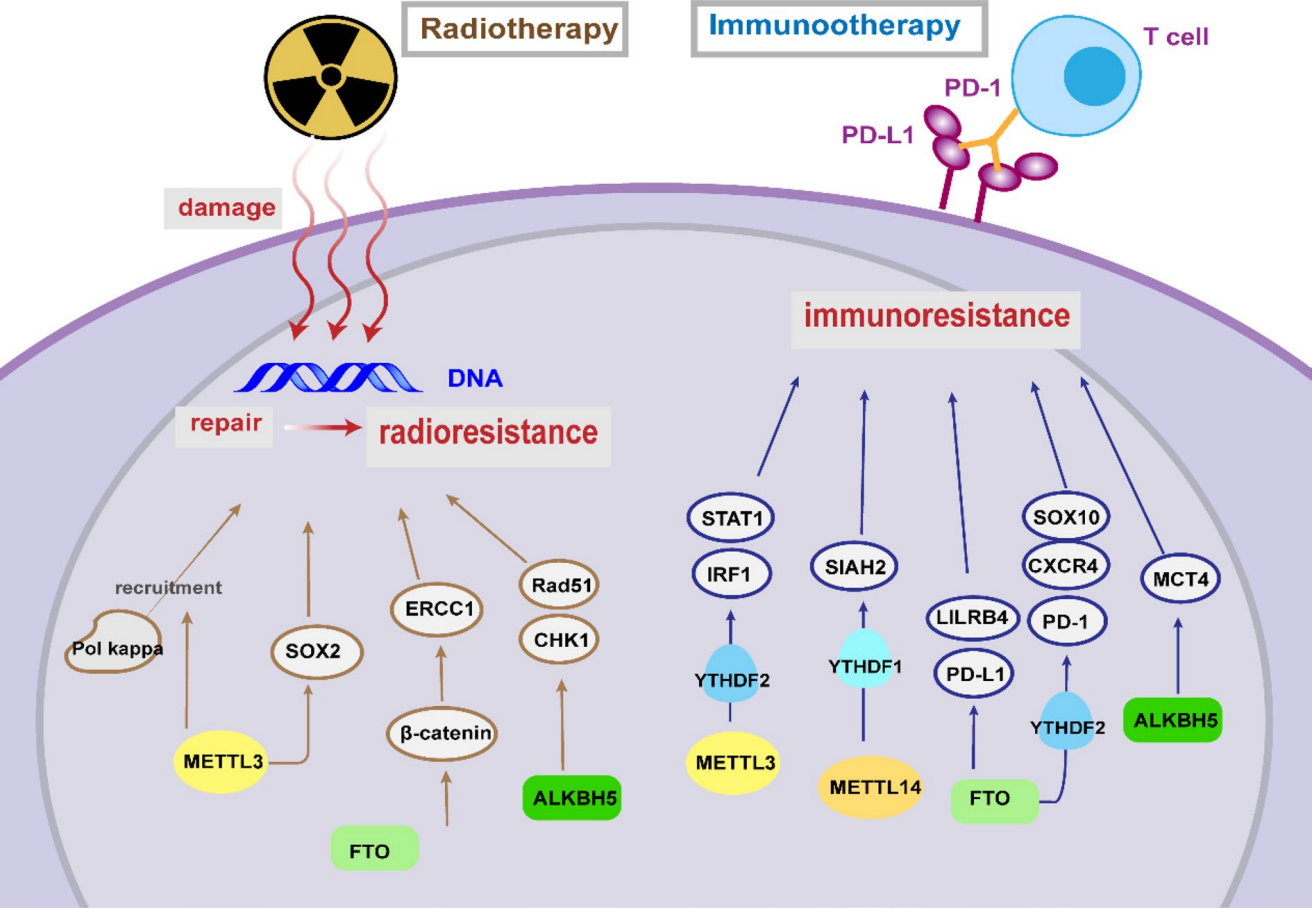
The role and molecular mechanisms of m6A regulators in radiotherapy and immunotherapy resistance

In cervical squamous cell carcinoma (CSCC), upregulated FTO could promote β-catenin expression via demethylating its transcripts and further positively regulate the excision repair cross-complementation group 1 (ERCC1), contributing to the resistance to cisplatin and irradiation [132]. Researchers speculated that highly expressed ALKBH5 in GBM stem cells promoted radiotherapy resistance via modulating homologous recombination (HR). Deficiency of ALKBH5 induced inhibition of checkpoint kinases 1 (CHK1) and recombinase Rad51, both of which are key players in DNA damage repair [133]. Furthermore, the researchers previously revealed that FOXM1 interacted with MELK to block radiotherapy response [134], and in the present study, IR-induced upregulation of FOXM1 level was attenuated by ALKBH5 inhibition, implicating other possible pathway of ALKBH5-mediated radio-resistance in GBM [133]. Taken together, these findings demonstrated a fundamental need for further exploring the regulatory roles in resistance to radiotherapy (Table 3, Fig. 3).

**Table 3:** Role of m6A regulator in radiotherapy and immunotherapy

Drug	Regulator	Role	Level	Cancer	Target	Effect	Refs.
irradiation	METTL3	Oncogene	High	GBM	Pol kappa	regulates its localization to DNA damage sites	[130]
irradiation	METTL3	Oncogene	High	GBM	SOX2	Increases stability of SOX2 mRNA	[131]
irradiation	FTO	Oncogene	High	CSCC	β-catenin	Promotes expression of β-catenin	[132]
irradiation	ALKBH5	Oncogene	High	GBM	CHK1, Rad51	Promotes expression of CHK1 and Rad51	[133]
Anti-PD1	METTL3/14	Oncogene	High	CRC	STAT1, IRF1	stabilizing the STAT1 and IRF1 mRNA via YTHDF2 [135]	[135]
Anti-PD1	METTL14	Suppressor	NA	CCA	SIAH2	Promotes the degradation of SIAH2 mRNA via YTHDF1	[136]
Anti-PD1	YTHDF1	Oncogene	NA	Melanoma	cathepsins	Promotes the translation of lysosomal cathepsins	[137]
Anti-PD1	FTO	Oncogene	High	Melanoma	PD-1, CXCR4, SOX10	Reduces the degradation of PD-1, CXCR4, and SOX10 mRNAs	[138]
Anti-PD1	FTO	Oncogene	High	CRC	PD-L1	Promotes expression of PD-L1	[139]
Anti-PD1	ALKBH5	Oncogene	NA	Melanoma	MCT4	Promotes expression of MCT4	[140]

## m6A and immunotherapy resistance

Immunotherapy is one of the most promising therapeutic methods, which aims at combating tumors by stimulating and enhancing the host immune system, including immune checkpoint blockade (ICB), cell therapy, cancer vaccine and so on [141]. However, some patients exhibit unfavorable responses to immunotherapy, which restricts the further applications and development. Mounting evidences suggested that m6A modification could modulate anti-tumor immunity and tumor microenvironment (TME), influencing the therapeutic response to immunotherapy (Table 3; Fig. 3).

## Immune checkpoint blockade

Among diverse immunotherapy strategies, ICB therapy is an unprecedented anti-tumor therapy by blocking inherent immune inhibiting factors, such as programmed cell death 1 (PD-1) or their ligands PD-L1, and cytotoxic T-lymphocyte antigen 4 (CTLA-4) [142]. Advances have been made in the clinical implements, such as PD-1 inhibitors pembrolizumab and nivolumab which are approved by FDA for advanced melanoma and NSCLC [143]. Nevertheless, for low‐mutation‐burden cancer patients, the failure of ICB or relapse are still common [144, 145]. Increasing researches revealed that m6A modification markedly affected the therapeutic resistance against ICB.

In mismatch‐repair‐proficient or microsatellite instability‐low (pMMR‐MSI‐L) tumors with low mutation burden, which constitutes about 85% of CRC, depletion of METTL3/14 could enhance ICB responses through increasing infiltration of cytotoxic CD8 + T cells and promoting secretion of IFN-γ, CXCL9, and CXCL10 in TME in vivo [135]. Mechanistically, METTL3/14 loss promoted IFN-γ-STAT1-IRF1 signaling through stabilizing the STAT1 and IRF1 mRNA via YTHDF2 [135]. Paradoxically, METTL14 was proposed to increase sensitivity to anti-PD1 immunotherapy in cholangiocarcinoma (CCA). METTL14 could enrich m6A in the 3'UTR of the siah E3 ubiquitin protein ligase 2 (SIAH2) mRNA and lead to YTHDF1-mediated decay. Therefore, deficiency of SIAH2 elevated PD-L1 expression level by reducing its K63-linked ubiquitination, and further sensitized tumor to ICB [136].

The previous study has confirmed that therapeutic efficacy of PD-L1 checkpoint blockade was enhanced in YTHDF1(−/−) mice. Depletion of YTHDF1 suppresses the translation of lysosomal cathepsins in DCs, downregulated cathepsins attenuates antigen degradation and thus enhances cross-presentation to CD8 + T cells, further increased IFN-γ expression in CD8 + T cells upregulates PD-L1 expression in tumor cells [137]. Accordingly, patients with low YTHDF1 level show more CD8 + T cell infiltration, implicating the potential of YTHDF1 inhibition in overcoming low response to ICB [137].

FTO was supposed to negatively regulate anti-PD-1 immunotherapy response in melanoma. Knockdown of FTO elevated the m6A level in transcripts of several critical melanoma-promoting genes, including PD-1, C-X-C motif chemokine receptor 4 (CXCR4), and SOX10, then accelerated RNA decay mediated by YTHDF2, restoring IFN-γ response in vitro and sensitizing anti-PD-1 treatment in vivo [138]. Moreover, Tsuruta et al. found that FTO could upregulate PD-L1 expression, positively regulating anti-PD-1 blockade response in CRC. Contrary to previous recognition that PD-L1 was upregulated by IFN-γ signaling [146], they indicated that FTO regulates PD-L1 expression in an IFN-γ-independent manner in HCT-116 cells [139].

Immune evasion resulting from upregulated immune checkpoint genes was deemed as the origin of hypomethylating agents (HMAs)-induced drug resistance [147]. HMAs, such as Azacitidine (AZA) and Decitabine (DAC), are wildly used in AML or myelodysplastic syndrome (MDS) treatment [148, 149]. Su et al. found that DAC treatment also cause reduction of global m6A abundance in AML cells, possibly related with the overexpressed FTO. They observed that FTO inhibition induced by small-molecule compounds, downregulated the immune checkpoint gene leukocyte immunoglobulin-like receptor subfamily B4 (LILRB4), with a greater tendency than PD-L1/2, further sensitized leukemia cells to T cell cytotoxicity and overcame HMA-induced immune evasion [150]. Although the low expression of PD-L1/2 in AML impedes application of anti-PD-1 blockade, these findings underlying the significance of combining FTO inhibitors or anti-LILRB4 agents and HMAs for myeloid malignancies. Moreover, FTO was supposed to mediated immune evasion via rewiring tumor glycolytic metabolism to restrict effector T cells functions. FTO-dependant demethylation facilitates the expression of several basic leucine zipper (bZIP) family transcription factors such as JunB and C/EBPβ, and then upregulates glycolysis-associated genes, which further establishes a metabolic advantage over T cells. Herein, inhibition of FTO promotes T cell infiltration and synergizes with anti-PD-L1 blockade in melanoma [151].

Via analyzing clinical data, ALKBH5 was identified as a predictive biomarker of anti–PD-1 blockade ressistance in melanoma. In mechanism, ALKBH5 deletion reduces MCT4 expression and lactate content in tumor interstitial fluids, which ultimately suppressed the Treg and polymorphonuclear myeloid derived cell [140]. In addition, the silico analysis showed that the m6A regulator ELAVL1 played a crucial role in the therapeutic response of anti-PD-L1 therapy in GBM [152]. In addition, a great deal of bioinformatic researches have underscored the close correlation between m6A methylation and immunotherapy response in various cancer [153–155]. For instance, via thoroughly analyzing the epigenetic modification patterns of 1518 GC patients, a subtype with high FTO level was identified to correlate with poor survival and immunotherapy resistance, which are potentially benefited from combination FTO inhibition and ICB [155].

## Implications and future directions

With growing studies underlying the mechanisms of how m6A modification influences cancer therapeutic resistance, targeting at m6A to optimize cancer therapy becomes increasingly appealing. There are several promising research directions refueling the scientific interests.

## Predictive biomarkers for therapy responses

More effective assessment approaches to predict potential therapeutic resistance and evaluate treatment effects have been demanded for a long time. As presented above, aberrant expressions of m6A regulators are ubiquitously involved in various resistance, providing theoretic fundament for predicting treatment responses. Amounting evidences identified that alteration of global m6A levels and regulators expression are closely related with therapy response, for example, upregulated METTL3 level was identified in TMZ resistant GBM tissue [64]. Besides next-generation sequencing (NSG) technology, tumor-derived organoids and network-based machine learning are also conducive to predicting drug efficiency or tumor resistance [156]. Recent years have witnessed plentiful mechanistic studies and bioinformatics analysis identifying the prognostic potential of m6A regulators in various cancer. However, there is still a long way from clinic applications.

## Targeting m6A regulators for overcoming resistance

Specific inhibitors of m6A regulators have demonstrated exciting anti-tumor effects, including inhibitors of METTL3, FTO, ALKBH5, IGF2BP1 [157], while the therapeutic effects of METTL3 activators remain unverified [158]. Especially, researches have underscored the potential of several specific inhibitors to overcome therapeutic resistance, supporting the combined applications with present therapeutics.

Rhein is the first natural FTO inhibitor which competitively binds to the catalytic domain of FTO and displays therapeutic efficacy in leukemia mice [159]. The NSAID, meclofenamic acid 2 (MA2) was found to inhibit FTO and thus suppress glioblastoma progression [160]. The small-molecule compounds CHTB and N-CDPCB were identified via crystal structure screening, providing new binding sites for targeting FTO [161, 162]. R-2-hydroxyglutarate (R-2HG) inhibits progression of AML in vitro an in vivo through inhibiting FTO, synergizing with a series of first-line chemotherapy agents such as all-trans retinoic acid (ATRA), Azacitidine, Decitabine, and Daunorubicin [86]. Also, R-HG displays growth-suppressive effects in GBM cell lines and cooperates with TMZ [86]. Subsequently, Huang et al. have reported that tricyclic benzoic acid FB23-2 could suppress the proliferation and enhance the differentiation/apoptosis of AML cells [163]. Su et al. identified that two small molecular inhibitors, CS1, and CS2, can sensitize leukemia cells to T cell cytotoxicity, with potential to overcome HMA-induced immune evasion [150]. For immunotherapy, another inhibitor Dac5 relived the constraints on T cells activation and functions imposed by FTO, which improves the ICB efficacy in melanoma [151]. Recent advances showed that the FB23 analog, FB23-13a exerted a stronger anti-proliferative effect on AML cells both in vitro and in vivo [164]. Recent researches made favorable progressions in more tumor types rather than AML and glioma. A small-molecule compound, 18097 significantly restrained growth and colonization of breast cancer cells [165]. Significantly, the oxetane class of FTO inhibitors, FTO-43 demonstrated anti-tumor potency comparable to 5-FU in GC, glioblastoma and AML models [166]. And Qin et al. proposed compound C6, a 1,2,3-triazole analogues, as an orally antitumor agent for esophageal cancer [167]. Additionally, the technologies also keep evolving. researchers have synthesized a hybrid FTO inhibitor by merging fragments from previously reported inhibitors with anti-leukemia activity [168]. FTO inhibitor-loaded GSH-bioimprinted nanocomposites (GNPIPP12MA) synergized GSH depletion to anti-leukemogenesis, which augmented the therapeutic efficacy of the PD-L1 blockade [169].

The initial small molecule METTL3 inhibitors were discovered via the high-throughput docking into the SAM binding site, which are indeed adenosine analogues [170]. However, due to the binding promiscuity and poor permeability, non-nucleoside inhibitors were developed. A small-molecule METTL3 inhibitor, UZH1a demonstrated potent growth inhibition of AML [171]. UZH2, the analogues of UZH1a, exhibits antitumor effects in AML and prostate cancer cell lines [172]. Remarkably, in vitro and in vivo experiments identified potent anti-leukemia efficacy of STM2457, without influencing normal hematopoiesis [173]. The specific ALKBH5 inhibitor ALK-04 was found to improve anti-PD-1 therapy efficiency in melanoma [140]. Selberg et al. discovered two compounds selectively inhibited ALKBH5, with anti-proliferative effects in specific leukemia cell lines [174]. Moreover, BTYNB was identified as a potent and selective IGF2BP1 inhibitor, exhibiting growth-suppressive effects in melanoma and OC cells [175].

**Fig. 4:**
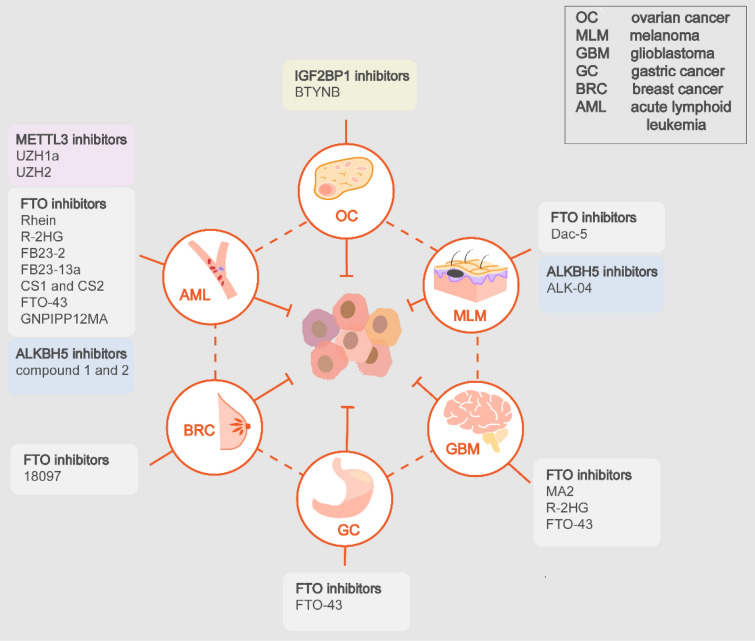
The applications of m6A regulator-targeted inhibitors in cancers

Collectively, m6A regulator-targeted drugs have gained lots of attention as a promising therapeutic strategy. For the specific inhibitors verified to improve therapeutic effects, further pre-clinical experiments should be boosted. For those novel durgs, their potentials to synergize present therapeutics and overcome resistance should be explored. Also, more endeavors should be put into developing more potent and selective inhibitors or activators (Table 4; Fig. 4).

**Table 4:** m6A regulator-targeted inhibitors

Target	Drug	Cancer	Effect	Ref.
METTL3	Adenosine analogues	NA	Reduces RNA m6A	[170]
	UZH1a	AML	Suppress proliferation and viability of tumor cells in vitro	[171]
	UZH2	AML, prostate cancer	More potent anti-proliferative effects in vitro	[172]
	STM2457	AML	Anti-leukemia efficacy in vitro and in vivo	[173]
FTO	Rhein	AML	Anti-leukemia efficacyin vitro and in vivo	[159]
	MA2	GBM	reduces GBM stem cell proliferation in vitro and tumor progression in mic	[160]
	CHTB	NA	Increases RNA m6A	[161]
	N-CDPCB	NA	Increases RNA m6A	[162]
	R-2HG	AML, GBM	Anti-leukemia efficacy in vitro and in vivo, suppresses GBM in viro, synergizes with chemotherapeutic drugs	[86]
	FB23-2	AML	Anti-leukemia efficacy in vitro and in vivo	[163]
	CS1 and CS2	AML	potent anti-leukemic efficacy in mouse models, sensitize leukemia cells to T-cell cytotoxicity, overcomes immune evasion	[150]
	Dac5	Melanoma	Promotes activation and effector state of T cell, improving anti-PD1 blockade effects	[151]
	FB23-13a	AML	Stronger anti-leukemia efficacy in vitro and in vivo	[164]
	18,097	Breast cancer	restrain in vivo growth and lung colonization	[165]
	FTO-43	GC, AML, GBM	Potent anti-tumor effects in mouse model	[166]
	Compound C6	Esophageal cancer	Anti-tumor efficacy in vitro and in vivo	[167]
		AML	Anti-leukemia and improves anti-PD1 blockade efficacy	[169]
ALKBH5	ALK-04	Melanoma	Improve anti-PD-1 therapy efficiency	[140]
	Compound 1 and 2	AML	Anti-proliferative effects in specific AML cell lines	[174]
IGF2BP1	BTYNB	Ovarian cancer	Anti-tumor efficacy in vitro and in vivo	[175]

## Potential of m6A regulation in immunotherapy

It has been widely recognized that m6A modifications can regulate the fate and biological behaviors of tumor cell. At the same time, m6A exhibits prominent influences on immune cells, providing opportunities for developing immunotherapy. Both METTL3 and YTHDF2 are positive regulators of NK cell functions, including maintaining the homeostasis, maturation, survival, anti-tumor and anti-viral activity. Researchers have successfully enhanced the proliferation and cytotoxicity of NK cells in vitro by modulating m6A regulators, providing support for adoptive NK cell-based immunotherapy, such as chimeric antigen receptor (CAR) NK cells and induced pluripotent stem cell (iPSC)-derived NK cells [176, 177]. For T cells, METTL3 exerts an essential role in regulating the homeostasis and differentiation [178], influencing the anti-tumor responses in a bidirectional complex manner. On the one hand, depletion of METTL3 facilitates anti-tumor immunity by restraining Treg cells, on the other hand, reduced METTL3 in CD4 + T cell impairs humoral immunity by suppressing the differentiation and functional maturation of T follicular helper cell [179, 180]. Additionally, tumor-intrinsic FTO could suppress the activation and effector states of CD8 + T cells [151]. For macrophages, METTL3 exerts positive control on its polarization and immune functions, ablating METTL3 can promote tumor growth and metastasis. Besides, METTL3 depletion in macrophages reduced the efficacy of PD-1 blockade therapy and levels of lipopolysaccharide (LPS)-induced TNF-α [181, 182]. Also, METTL14 was found to regulate the functions of immunomodulatory ligands in macrophages, and METTL14 deficiency in macrophage1919s inhibited the anti-tumor function of CD8 + T cells and promoted tumor growth [183]. Taken together, these findings highlight the significant roles of m6A regulators in immune cells.

Furthermore, m6A modifications have the promising potential to remodel the TME, influencing cancer progression and responsiveness to immunotherapy. METTL3 plays a dual role as either an oncogene or a tumor suppressor gene in different types of cancers, with upregulated or downregulated expression. To complicate matters further, the level of METTL3 was significantly related with infiltration of various immune cells, in positive or negative correlation. For example, downregulation of METTL3 in testicular germ cell tumors (TGCT) tissue is positively correlated with levels of tumor-infiltrating CD8 + T cells, CD4 + T cells, and NK cells [184], whereas the increased METTL3 level in CRC is negatively related with infiltration of CD8 + T cells and secretion of IFN-γ, CXCL9, and CXCL10 [135]. Such complicated situation also exists in several other m6A regulators, such as METTL14, ALKBH5, and YTHDFs [185]. Significantly, except for affecting infiltration of immune cells, m6A exerts influences on TME by regulating the expression of immune checkpoint genes. For example, depletion of FTO induces suppression of LILRB4 in AML [150], and ALKBH5 facilitates expression of PD-L1 in intrahepatic cholangiocarcinoma (ICC) [186].

Overall, the above studies emphasize the potential of targeting m6A modifications to enhance the efficacy of current immunotherapy, as well as to develop novel strategies. Developing specific inhibitors or activators is undoubtedly meaningful. As mentioned above, agents capable of improving anti-tumor immunity have already been reported [150, 151], and the combined application has been verified to sensitize tumor cells to ICB [138, 140]. Additionally, modulating m6A modifications appears as a promising direction in adoptive cell therapy, including CAR NK cells, iPSC-derived NK cells and CAR T cell therapy. Since nanoparticles (NPs) have emerged as promising carriers in cancer precision therapy [187], targeted delivery of NP-encapsulated m6A regulators or target genes into TAMs could be a research direction of great value.

## Discussion

The regulatory roles of m6A modification in controlling cancer therapeutic response/resistance have gained increasing attention in recent years. Significantly, targeting m6A modification system is highly conducive to improve cancer therapy efficacy. Compared with existing review, our study is much more comprehensive and systematic, not only supplementing the latest progresses, but also compensating the shortcomings of previous studies. Resistance to chemotherapy, targeted therapy, radiotherapy and immunotherapy were respectively overviewed, with adequate attention paid on immunotherapy. Furthermore, the clinical implications of m6A were highly valued to prevent resistance and develop new therapeutic strategies.

However, with breakthroughs made in this field, there are still contradictions and uncertainties in relevant rationales: (i) in the same cancer, the expression of m6A writers and erasers are regulated in the same direction, leading to discrepancy in m6A concentration alteration. For instance, both METTL3 and FTO are upregulated in NSCLC tissue. Liu et al. have reported significantly overexpressed METTL3 [116], while another research showed increased FTO expression and decreased m6A level in the serum exosomes [118]; (ii) in the same cancer, m6A writers and erasers display similar effects on therapy resistance. Take the resistance to TMZ in GBM as example, it was demonstrated that METTL3 contributed to the resistance by enhancing DNA damage repair [63]. Intriguingly, FTO was also reported to enhance the resistance via protecting PDK1 mRNA from degradation [65]; (iii) for the same type of regulators, they have opposite regulatory roles for the same therapy. For instance, METTL3 and METTL14 were reported to regulate sorafenib resistance of HCC reversely. METTL3 exerts positive regulation by inhibiting OATP1B1 and OATP1B3 expression to decrease sorafenib uptake [103]. On the contrary, METTL14 induced transactivation of OATP1B1 and OATP1B3 to sensitize HCC cells [106]; (iv) for the same regulator, it has contrary influences on the same therapy. Represented by the most learned METTL3, it was revealed that METTL3 promoted resistance to cisplatin by stimulating AKT1 in NSCLC [35]. However, Ling et al. found that METTL3 participated in the re-sensitization to cisplatin mediated by propofol [36].

Thus, in order to clarify these uncertainties and deepen the understanding of therapeutic resistance, more researches of m6A regulation are required. There are some reasonable approaches, including: (1) developing a precise and efficient editing tool to fine-tuning m6A modification of specific targets, a gene, a group of cells and even a specific m6A stie; (2) exploring the target selectivity of m6A regulators in different cellular context, different cancer cells from different tissue origins; (3) developing highly selectively m6A-targeted agents and exploring the feasibility of combined application; (4) mining the potential of m6A modifications in immunotherapy, developing targeted agents to activate endogenous anti-tumor immunity and remodel TME.

## Conclusion

In virtue of the ubiquitous regulation of m6A on the RNA fates, m6A modification plays s significant role in regulating cancer therapeutic resistance, which lays the foundation for circumventing resistance and optimizing cancer treatment. Although numerous advancements have been achieved in exploring the underlying mechanisms and clinic implications in this field, our knowledge is in infancy. New approaches and techniques, more large-scale and deeper researches are required, and more attention should be given to the potential of emerging immunotherapy.

## Data Availability

Not Applicable.

## Abbreviations

5-Fu: 5-Fluorouracil
ALKBH3/5: AlkB homolog 3/5 RNA demethylase
AKR1C1: Aldo–keto reductases 1C1
AML: Acute myeloid leukemia
APNG: Alkylpurine–DNA–N-glycosylase
AKT1: AKT serine/threonine kinase 1
ATP7A: ATPase copper transporting alpha
ATRA: All-trans retinoic acid
BC: Breast cancer
BRCA1: Breast cancer type 1
BCRP: Breast cancer resistance protein
CAFs: Cancer-associated fibroblasts
CAR: Chimeric antigen receptor
CBSLR: CBS mRNA-destabilizing lncRNA
CCA: Cholangiocarcinoma
CDA: Cytidine deaminase
CDS: Coding sequence
CHK1: Checkpoint kinases 1
CRC: Colorectal cancer
CSCC: Cervical squamous cell carcinoma
CTLA-4: Cytotoxic t-lymphocyte antigen 4
CTX: Cetuximab
CXCR4: C-X-C motif chemokine receptor 4
DDP: Cisplatin
DUSP6: Dual-specificity phosphatases 6
DGCR8: DiGeorge Syndrome Critical Region 8
DOX: Doxorubicin
DNMT3a/b: DNA methyltransferase-3a/b
DYRK1B: Dual-specificity Y-phosphorylation-regulated kinase 1B
E2F1: E2F transcription factor 1
EGFR: Epidermal growth factor receptor
eIF: Eukaryotic initiation factor
ERCC1: Excision repair cross-complementation group 1
ERRγ: Estrogen receptor related receptor γ
FAO: Fatty acid β-oxidation
FMRPs: Fragile X mental retardation proteins
FOXM1: Forkhead box M1
FSP1: Ferroptosis suppressor protein 1
FTO: Fat mass and obesity-associated protein
GC: Gastric cancer
GADD45A/B: DNA damage inducible alpha/beta
GBM: Glioblastoma
GLS1: Glutaminase 1
HGF: Hepatocyte growth factor
HCC: Hepatocellular carcinoma
HuR: Human antigen R
IGF2BP: IGF2 mRNA binding protein
JAK2: Janus kinase 2
LATS2: Large tumor suppressor kinase 2
LC: Liver cancer
LILRB4: Leukocyte immunoglobulin-like receptor subfamily B4
LGR5: Leucine rich repeat containing G protein-coupled receptor 5
m6A: N6-methyladenosine
MALAT1: Metastasis associated lung adenocarcinoma transcript 1
MDR1: Multidrug resistance protein
METTL3/14/16: Methyltransferase-like 3/14/16
MGMT: O6-methylguanine–DNA methyltransferase
MRE11: Meiotic recombination 11 homolog 1
MSCs: Mesenchymal stem cells
MTC: Methyltransferase complex
NANOG: Nanog homeobox
NF-κB: NF-kappaB
NSCLC: Non-small cell lung cancer
NPC: Nasopharyngeal carcinoma
OATP1B: Organic anion-transporting polypeptide 1B
OC: Ovarian cancer
OSCC: Oral squamous cell carcinoma
PARPi: Poly(ADP-ribose) polymerase inhibitors
OXA: Oxaliplatin
PC: Pancreatic cancer
PDK1: Phosphoinositide dependent kinase-1
PD-1: Programmed cell death 1
PDAC: Pancreatic ductal adenocarcinoma
PDGFR-β: Platelet-derived growth factor receptor
PTEN: Phosphatase and tensin homolog
NF-κB: RBM15/15B: RNA-binding motif protein 15/15B
R-2HG: R-2-hydroxyglutarate
RAP1: Ras-associated protein1
SIAH2: Siah E3 ubiquitin protein ligase 2
SOX4: SRY-box transcription factor 4
SRSF3: Serine/arginine-rich splicing factor 3
STAT3: Signal transducer and activator of transcription 3
Tax: Taxol
TKI: Tyrosine kinase inhibitor
TMZ: Temozolomide
TRAF: Tumor necrosis factor receptor-associated factors
TRIM29: Tripartite motif-containing protein
TFAP2C: Transcription factor-activating enhancer-binding protein 2C
VEGFR: Vascular endothelial growth factor receptor
VIRMA/KIAA1429: Virlike m6A methyltransferase-associated
WIF-1: Wnt inhibitory factor 1
WTAP: WT1-associated protein
XLF: XRCC4-like factor
YTHD: YTH domain family of protein
ZC3H13: Zinc finger CCCH-type containing 13
γH2AX: Gamma histone variant H2AX
